# Insonation of Systemically Delivered Cisplatin-Loaded Microbubbles Significantly Attenuates Nephrotoxicity of Chemotherapy in Experimental Models of Head and Neck Cancer

**DOI:** 10.3390/cancers10090311

**Published:** 2018-09-05

**Authors:** Hang-Kang Chen, Shu-Mei Zhang, Junn-Liang Chang, Hsin-Chien Chen, Yi-Chun Lin, Cheng-Ping Shih, Huey-Kang Sytwu, Mei-Cho Fang, Yuan-Yung Lin, Chao-Yin Kuo, Ai-Ho Liao, Yueng-Hsiang Chu, Chih-Hung Wang

**Affiliations:** 1 Graduate Institute of Medical Sciences, National Defense Medical Center, Taipei 11490, Taiwan; hwalongchen@yahoo.com.tw (H.-K.C.); lyc_1023@yahoo.com.tw (Y.-C.L.); yking1109@gmail.com (Y.-Y.L.); 2 Department of Otolaryngology-Head and Neck Surgery, Tri-Service General Hospital, National Defense Medical Center, Taipei 11490, Taiwan; acolufreia@yahoo.com.tw (H.-C.C.); zhengping_shi@yahoo.com.tw (C.-P.S.); chefsketchup@hotmail.com (C.-Y.K.); 3 Graduate Institute of Biomedical Engineering, National Taiwan University of Science and Technology, Taipei 10607, Taiwan; m1482073@gmail.com; 4 Department of Pathology, Taoyuan Armed Forces General Hospital, Taoyuan 32551, Taiwan; junn9liang@yahoo.com.tw; 5 Department of Biomedical Engineering, Ming Chuan University, Taoyuan 33348, Taiwan; 6 Graduate Institute of Microbiology and Immunology, National Defense Medical Center, Taipei 11490, Taiwan; sytwu@ndmctsgh.edu.tw; 7 Laboratory Animal Center, National Defense Medical Center, Taipei 11490, Taiwan; misa@mail.ndmctsgh.edu.tw; 8 Department of Biomedical Engineering, National Defense Medical Center, Taipei 11490, Taiwan; 9 Taichung Armed Forces General Hospital, Taichung 41168, Taiwan

**Keywords:** ultrasound, microbubble, cisplatin, nephrotoxicity, head and neck cancer, chemotherapy

## Abstract

The use of cisplatin (CDDP), the most common chemotherapy drug for head and neck cancer, is limited by its undesirable side effects, especially nephrotoxicity. We investigated ultrasound microbubbles (USMB) as a tool to increase the local intra-tumoral CDDP level while decreasing systemic CDDP cytotoxicity. We allowed CDDP to interact with human serum albumin and then sonicated the resulting CDDP‒albumin complex to generate CDDP-loaded MBs (CDDP-MBs). We then established a head-and-neck tumor-bearing mouse model by implanting FaDu-fLuc/GFP cells into severe combined immunodeficiency mice and used IVIS^®^ bioluminescence imaging to determine the tumor xenograft formation and size. Twice weekly (until Day 33), we administered CDDP only, CDDP + MBs + US, CDDP-MBs, or CDDP-MBs + US intravenously by tail-vein injection. The US treatment was administered at the tumor site immediately after injection. The in vivo systemic distribution of CDDP indicated that the kidney was the most vulnerable organ, followed by the liver, and then the inner ear. However, CDDP uptake into the kidney and liver was significantly decreased in both the CDDP-MBs and CDDP-MBs + US groups, suggesting that MB binding significantly reduced the systemic toxicity of CDDP. The CDDP-MBs + US treatment reduced the tumor size as effectively as conventional CDDP-only chemotherapy. Therefore, the combination of CDDP-MBs with ultrasound is effective and significantly attenuates CDDP-associated nephrotoxicity, indicating a promising clinical potential for this approach.

## 1. Introduction

Squamous cell cancer of the head and neck (SCCHN) is the sixth most common type of cancer worldwide, with more than 600,000 new patients diagnosed annually [1]. Head and neck cancers are classified by the area they develop within paranasal sinuses, nasal cavity, oral cavity, pharynx, larynx, and salivary glands. Treatments for head and neck cancers include surgery, radiation therapy, chemotherapy, targeted therapy, or a combination of treatments, depending on the location and size of the tumor and the risk of recurrence. Current surgery or radiation therapy achieves five-year survival rates of around 70–90% in patients with early stage I and II tumors, but almost two-thirds of patients with SCCHN present with advanced stages at diagnosis [2]. In this situation, treatment with chemotherapy, singly or in combination with other cancer treatments, is usually inevitable. 

Chemotherapy based on systemic cis-diaminedichloroplatinum (cisplatin, CDDP) is a time-honored treatment for head and neck cancer. However, cisplatin shows no specificity or selectivity for neoplastic over non-neoplastic cells, so that increasing cumulative doses of cisplatin can result in unwanted side effects that include nephrotoxicity, hepatotoxicity, hematologic toxicity, and hearing loss. Among these side effects, ototoxicity-related hearing impairment usually manifests as irreversible, progressive, and bilateral sensorineural hearing loss that greatly impairs the patient’s quality of life [3]. Similarly, hematologic adverse effect and nephrotoxicity can result in life-threatening sepsis and renal failure. Thus, ongoing research is increasingly aimed at developing new strategies to maximize the cisplatin concentration in tumors while minimizing the cisplatin levels in normal tissues [4,5,6,7,8]. 

One strategy that shows promise is the use of microbubbles (MBs), which are presently being investigated as carriers for drugs, small molecules, nucleic acids, and proteins for delivery to ultrasound-targeted locations [9,10,11]. Originally developed as ultrasound contrast agents for imaging and diagnostics, microbubbles (MBs) are air-core bubbles wrapped by a lipid, protein, or polymer shell and typically have a size range of 1–8 μm. Upon exposure to ultrasound, MBs may exhibit several physical phenomena, including oscillation, cavitation, or sonoporation, which cause transient and reversible changes in membrane permeability in surrounding cells. Typically, simple and repetitive contraction and expansion of MBs in response to an ultrasound wave (stable cavitation) can create micro-streaming around the MBs and induce shear stress, which then acts on cell membranes and leads to ion channel/receptor modulation and alterations in cell membrane permeability [12]. A related phenomenon, termed inertial cavitation, is caused by sudden expansion and then rapid collapse of the MBs. This creates a strong mechanical stress, similar to a shockwave or micro-jet, which reacts at sonic speed with the cell surface and can even create holes in blood vessels and cell membranes [13,14,15]. This mechanical action of ultrasound and microbubbles is exploited with the so-called ultrasound-microbubble (USMB) technique to increase the permeability of biological barriers to drugs, genes, and their carriers, and it has opened up a wide range of applications for drug and gene delivery to sites of action, including tumors [16]. 

One of the most promising targets for USMB-aided chemotherapy is head and neck cancer, because most of these cancers and their cervical metastatic nodes are clinically accessible. Malignancies of the upper aerodigestive tract usually invade structures that are vital for the functions of speech, mastication, and swallowing, as well as great vessels. The associated morbidity of these malignancies can therefore have a substantial impact on the patient’s quality of life. In the case of SCCHN, the concept of organ preservation through the use of combined chemotherapy and radiation therapy has become a crucial aspect of therapy evaluation over the past two decades [17]. Multiple strategies and various agents have been developed to substitute for CDDP in attempts to lessen the cytotoxicity and systemic side effects, but no current treatment has yet shown superiority to CDDP-based chemoradiation for locally advanced SCCHN [18]. 

The aim of the present study was to evaluate the feasibility of using USMB as a tool to increase the local intra-tumoral CDDP level, while decreasing systemic CDDP cytotoxicity in an experimental animal model of head and neck cancer. 

## 2. Results

### 2.1. Assessment of Binding Efficiency CDDP-Loaded Albumin MBs

**Figure 1:**
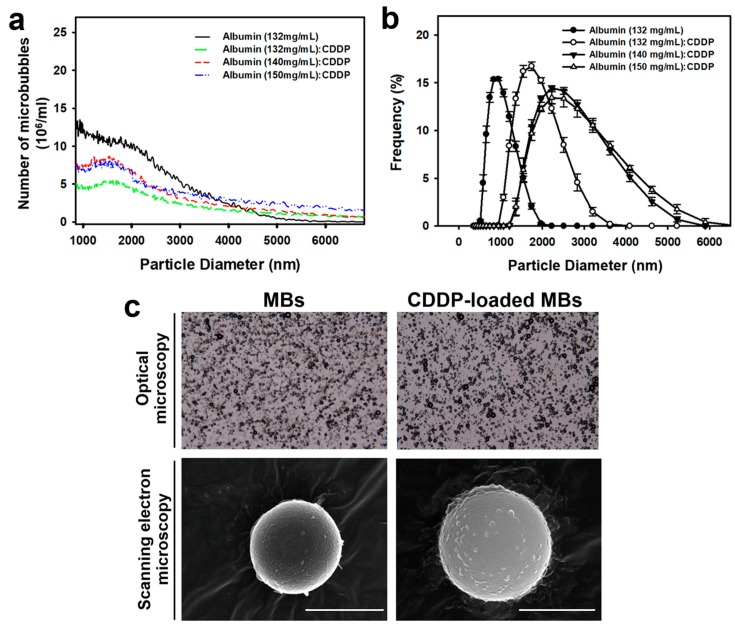
Quantification and light and scanning electron microscopy images of unloaded MBs (MBs) and cisplatin-loaded MBs (CDDP-MBs). (**a**) The number-dependent and (**b**) frequency-dependent size distribution of various MBs. Values are expressed as mean ± SEM (*n* = 5); (**c**) Representative light microscopy and scanning electron microscopy images of MBs and CDDP-MBs. Scale bar = 2 μm.

As shown in Table 1 and Figure 1, the average size and concentration of laboratory-prepared albumin MBs was 1.02 ± 0.11 μm and 1.40 ± 2.22 × 10^8^ microbubbles/mL, respectively. However, during preparation of the CDDP-loaded MBs (CDDP-MBs), the MB concentration would drop to 50% of its initial value when 6 mg/mL CDDP was added (Figure 1a). Increasing the concentration of albumin from 132 to 140 mg/mL, increased both the loading efficiency and the MB concentration, to 10.09 ± 0.23% and 1.09 ± 1.31 × 10^8^ bubbles/mL, respectively. Further increasing the concentration of albumin up to 150 mg/mL to mix with constant dosage of CDDP (6 mg/mL) led to a significant decrease in the loading efficiency to 7.70 ± 1.21%. Mixing increasing concentrations of CDDP (1, 2, 4, 6, and 12 mg/mL) with albumin (140 mg/mL) showed a dose-dependent efficacy of CDDP between 1 and 6 mg/mL, but no significant difference was noted between 6 and 12 mg/mL (Figure S1). Therefore, albumin at concentration of 140 mg/mL and CDDP at 6 mg/mL were used for preparation of CDDP-MBs for all subsequent experiments. 

The mean diameters of the CDDP-MBs ranged from 1.02 to 2.45 µm (Figure 1b,c), which were smaller than the CDDP-loaded albumin mesospheres published by Lee et al. [19]. 

### 2.2. Influence of MBs and US on In Vitro CDDP Cytotoxicity

**Figure 2:**
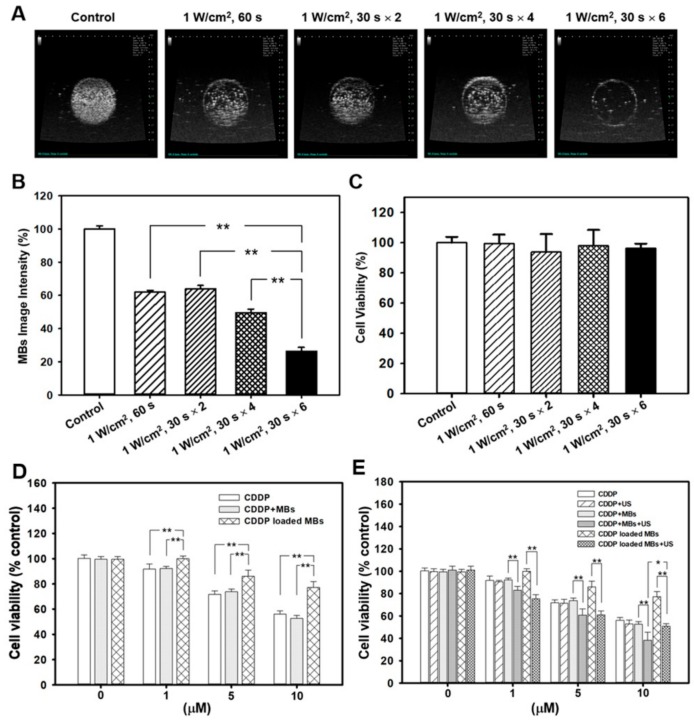
Destruction of ultrasound-targeted MBs and the comparison of in vitro CDDP toxicity on the FaDu cell line in various compositions of CDDP and MBs. (**A**) MB images and (**B**) the corresponding image intensities in various groups of acoustic exposure settings; (**C**) Percentage of cell viability evaluated by WST-1 analysis on FaDu cells 48 h after various ultrasound-targeted MB destruction treatments; (**D**) Cell viability was assayed by WST-1 after treatment with CDDP, CDDP + MBs, and CDDP-MBs for 48 h; (**E**) The capacity of the CDDP only, CDDP + MBs, and CDDP-MBs treatments to kill FaDu cells, with or without ultrasound exposure, was evaluated after 48 h of treatment by analyzing the cell viability using the WST-1 assay. Values are expressed as mean ± SEM (*n* = 5). * *p* < 0.05, ** *p* < 0.005.

The optimal ultrasound exposure parameters for MB destruction in vitro were examined by varying the settings of ultrasound exposure. At an acoustic intensity of 1 W/cm^2^, the MBs were destroyed an exposure-time–dependent manner, with 36.1%, 50.4%, and 73.7% destruction occurring at exposure times of 30 s × 2, 30 s × 4, and 30 s × 6, respectively, as demonstrated by MATLAB program-processed MB images (Figure 2A,B). Figure 2C shows that, at an acoustic intensity of 1 W/cm^2^, an ultrasound exposure of 30 s for 6 cycles did not affect the viability of FaDu cells in culture. Consequently, the ultrasound exposure of 30 s for six cycles was used for subsequent in vitro experiments.

We compared the cytotoxicity of CDDP and CDDP-MBs by treating 2 × 10^4^ FaDu cells/well in 24-well plates with various concentrations (1, 5, and 10 μM) of CDDP or CDDP-MBs, with or without ultrasound exposure. As shown in Figure 2D, without ultrasound exposure, the combination of MBs and CDDP showed no significant increase in toxicity compared with CDDP alone. However, CDDP-MB group showed a significantly lower toxic response than the CDDP or CDDP + MBs groups at any conditions of equal concentration (*p* < 0.005). This suggests that additional administration of MBs did not interfere with CDDP action, while CDDP, once incorporated into MBs, showed a significant reduction in cytotoxicity.

The application of ultrasound (1 W/cm^2^, 30 s for six cycles) also had no significant adverse effects on cell viability in the presence or absence of MBs (Figure 2E). Interestingly, the toxicity of CDDP was markedly enhanced after ultrasound treatment when MBs were co-administered than in the absence of MBs (CDDP + MBs + US vs. CDDP + MBs, *p* < 0.005; CDDP-MBs + US vs. CDDP-MBs, *p* < 0.005). In addition, the cytotoxicity was significantly higher for the 10 μM CDDP + MBs + US treatment than the CDDP-MBs + US treatment. Overall, these data suggest that ultrasound exposure of CDDP and MBs would enhance the cellular sensitivity to or uptake of CDDP, whereas when CDDP was bound to MBs (CDDP-MBs), this diminished the in vitro cytotoxicity (CDDP + MBs + US vs. CDDP-MB + US, *p* < 0.05).

### 2.3. The Combination of MBs and US Alters Systemic CDDP Uptake In Vivo

**Figure 3:**
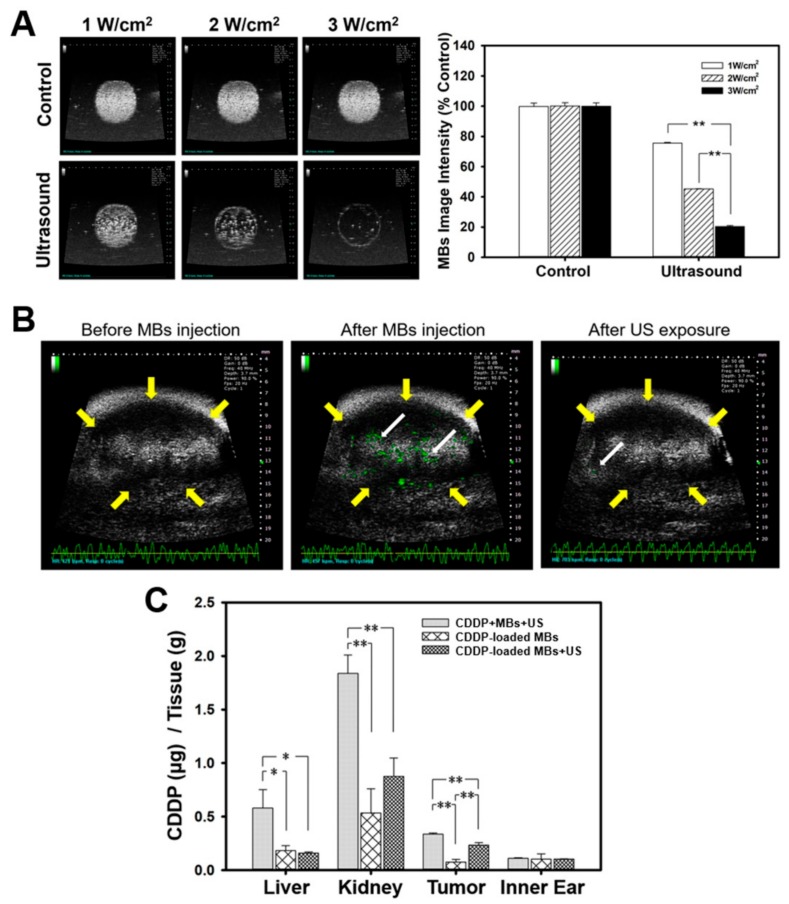
Influence of MBs and US on the in vivo CDDP accumulation. (**A**) High frequency US images of albumin MBs in tissue-mimicking agarose phantoms before (control) and after sonication at 1, 2, and 3 W/cm^2^ for 30 s. The image intensities of MBs are quantified; (**B**) A series of high-frequency US images that focus on head and neck carcinoma tumor lesions (yellow arrows) showed the presence of MBs (green) within the tumor (white arrows) after their administration via the mouse tail vein, and the disappearance of MBs when after exposure to therapeutic US for 30 s; (**C**) The CDDP level in vital organs was determined 20 min after a single intravenous administration of chemotherapy using ICP-MS quantitative method. Values are expressed as mean ± SEM (*n* = 5). * *p* < 0.05, ** *p* < 0.005.

The in vivo USMB parameters were optimized by testing different settings of acoustic intensity in tissue-mimicking agarose phantoms. The destruction efficiencies of albumin MBs at acoustic intensities of 1, 2, and 3 W/cm^2^ for 30 s were 24.39 ± 0.46%, 54.74 ± 0.1%, and 79.6 ± 0.64%, respectively (Figure 3A). We therefore set the US power at 3 W/cm^2^ and exposure at 30 s for all subsequent in vivo experiments. 

In vivo high frequency US images demonstrated the real time appearance of MBs within the tumor after tail vein MB injection, and this was followed by destruction of MBs at the tumor site by US irradiation at power of 3 W/cm^2^ and the subsequent disappearance of MBs after US exposure for 30 s (Figure 3B). These images imply that intravenous injected MBs circulated to the tumor lesions, where they could be destroyed by application of US exposure, thereby releasing the CDDP from CDDP-MBs. 

We therefore designed various CDDP-based chemotherapy regimens (CDDP only, CDDP + MBs, and CDDP-MBs), with or without the US exposure, for treatment of animal models of HNSCC. Figure 3C shows the systemic biodistribution of CDDP to relevant organs 20 min after administration of various CDDP-related chemotherapy treatments. Examination of the CDDP level in vital organs revealed that the kidney accumulated the greatest amount of CDDP among the organs investigated, followed by the liver and then the inner ear. Both the CDDP-MB and CDDP-MBs + US treatments significantly decreased the CDDP uptake in the kidney and liver when compared with CDDP administration alone, suggesting that conjugation of CDDP with MBs significantly reduces its systemic toxicity. These in vivo data were consistent with the in vitro experimental results in FaDu cells. No significant treatment differences were noted for the inner ear. 

The CDDP level in tumors were more than three times higher for the CDDP-MBs + US treatment than for the CDDP-MBs treatment (0.34 ± 0.07 vs. 0.09 ± 0.03, *p* < 0.005). These findings encouraged us to investigate the in vivo therapeutic outcome in an experimental head and neck cancer model treated with USMB and CDDP-MB-mediated chemotherapy for 33 days.

### 2.4. Chemotherapy with CDDP-MBs Provides Effective Treatment for Head and Neck Cancer 

**Figure 4:**
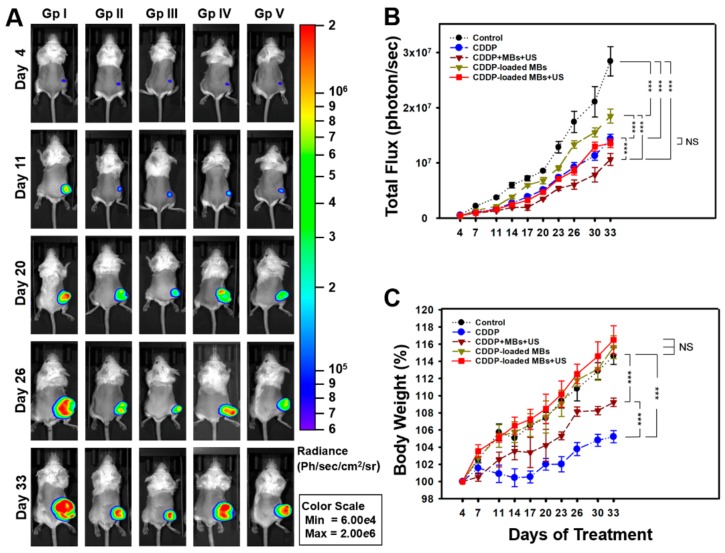
In vivo imaging system to monitor tumor growth and chemotherapy outcomes in living mice. (**A**) FaDu cells stably expressing luciferase and GFP reporter genes (FaDu-fLuc/GFP) were subcutaneously implanted into SCID mice. Four days later, the xenograft tumor-bearing mice were divided to five groups (*n* = 5 mice per group) including control (saline; Group I) and CDDP-based treatment groups: CDDP only (Group II), CDDP + MBs + US (Group III), CDDP-loaded MBs (CDDP-MBs; Group IV), and CDDP-loaded MBs + US (CDDP-MBs + US; Group V). Mice were repeatedly imaged to record luminescence signals and data were reported as radiance (photons/sec/cm^2^/steradian) with a color bar. A representative mouse from each of the five groups is shown; (**B**) Analysis of the IVIS images. Tumor size reductions at the end of treatment (day 33) in each chemotherapy group were 53.02 ± 0.61% (Group II), 62.54 ± 3.83% (Group III), 35 ± 4.46% (Group IV), and 52.29 ± 2.09% (Group V); (**C**) Effects of 33 days of chemotherapy on body weight gains. Values are expressed as mean ± SEM (*n* = 5). *** *p* < 0.0001 (two-way ANOVA with Tukey’s multiple comparison test). NS = not significant; Gp = group.

The anti-tumor effect of CDDP-based chemotherapy was evaluated in vivo on the growth of head and neck cancer by twice-weekly recordings of luciferase bioluminescence data derived from IVIS images (Figure 4A,B). On Day 33 after chemotherapy, all treatment groups showed significantly suppressed tumor growth compared to the control (Tukey’s multiple comparison test, *p* < 0.0001), with 30–65% reduction in tumor growth. Tumor reduction in the treatment groups between CDDP alone (Group II; 53.02 ± 0.61%) and CDDP-MBs + US (Group V; 52.29 ± 2.09%) did not differ significantly (*p* = 0.995); however, they all displayed significantly better outcomes compared to CDDP-MBs without ultrasound exposure (Group IV; 35 ± 4.46% tumor reduction). The combinations of CDDP, MBs, and US (Group III; 62.54 ± 3.83% tumor reduction) achieved the best inhibition of tumor growth among all treatments. This combination gave significantly better outcomes than Group II treated with CDDP alone (*p* < 0.0001) or Group V with CDDP-MBs plus ultrasound (*p* < 0.0001), indicating that CDDP combined with USMB can additively enhance in vivo uptake of CDDP into head and neck cancer cells. These data are not only consistent with our in vitro observations (Figure 2E), but are also supported by other recent studies [4,5,7]. 

Over the 33-day treatment period (Figure 4C), although all animals showed different degrees of gain in body weight (BW), the CDDP alone (Group II) and the CDDP + MBs + US (Group III) treatments both showed statistically smaller BW gain when compared with the control (Group I), CDDP-MBs (Group IV), or CDDP-MBs + US (Group V) treatments. The lower BW gain in Group III (CDDP + MBs + US) compared with Group II (CDDP alone) implies that the addition of albumin MBs may have a positive impact on the gain of BW during CDDP chemotherapy. 

### 2.5. Histological Examinations of CDDP-Based Chemotherapy

**Figure 5:**
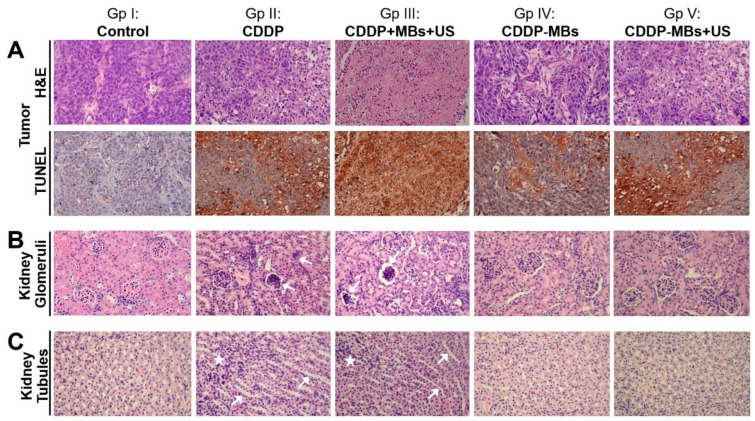
Effects of various CDDP-based chemotherapies on cell death in target tumor tissues and non-target kidney parenchyma. FaDu xenografted tumors and kidneys of SCID mice were removed for histological examination after various CDDP-based chemotherapies for 33 days. (**A**) Xenografted tumors were examined by H&E staining and TUNEL assays (original magnification ×400). Marked tumor cell apoptosis was observed in response to the CDDP + MBs + US (Group III) treatment, and to a lesser extent to the CDDP (Group II) and CDDP-MBs + US (Group V) treatments, which both exhibited a similar TUNEL positive staining. The CDDP-MBs group (Group IV) showed less positive staining; (**B**) CDDP-induced nephrotoxicity, which included congested, shrunken, and degenerated glomeruli (arrows); and (**C**) necrosis of renal tubular cells and tubular dilatation (arrows) associated with increased leukocyte infiltration (asterisks) were observed following CDDP (Group II) and CDDP + MBs + US (Group III) treatments.

**Figure 6:**
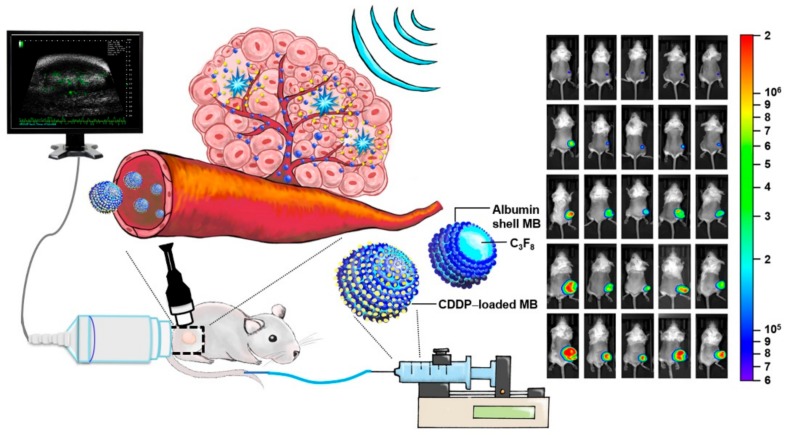
A working model of the gas-filled and CDDP-carrying MBs for the application of USMB in head and neck cancer systemic chemotherapy.

We used histopathological examinations to investigate the impact of various CDDP-based chemotherapies on tumor and kidney tissues at Day 33. The H&E-stained sections of tumor lesions showed that cancer cells exhibited a nest-like distribution and disordered arrangement (Figure 5). In the control group, tumor cells were closely arranged with complete and atypical structures. By contrast, the chemotherapy groups showed more tumor cell apoptosis, characterized by incomplete cell membranes, condensed cytoplasm, pyknotic or cracking nuclei, and cavity-shaped organization. Interestingly, the degree and extent of tumor cell apoptosis assessed by TUNEL staining among groups was consistent with the results of tumor reduction, as shown in Figure 6, as Group III (CDDP + MBs + US treatment) showed the greatest amount of apoptosis, whereas Group IV (CDDP-MB treatment) showed less apoptosis (Figures 5A and S2). Both the CDDP (Group II) and CDDP-MBs + US (Group V) treatments showed similar TUNEL positive staining. These results suggest that CDDP, when delivered in a bound form with MBs and exposed to ultrasound, can achieve a similar tumor killing effect to that achieved with CDDP alone.

The systemic cytotoxicity of CDDP chemotherapy in the non-target kidney tissue showed that CDDP treatment resulted in notable histological changes in the renal parenchyma, as indicated by vacuolation, or even atrophy, of the endothelium lining the glomerular tufts (Figure 5B) and necrosis and vacuolation of the epithelial lining and cystic dilation of the renal tubules (Figure 5C). Marked cytotoxic histological changes were noted in the kidneys of groups administered CDDP alone (Group II) or co-administered CDDP and MBs (Group III); however, these changes were attenuated in groups treated with CDDP-MBs (Group IV) or CDDP-MBs + US (Group V). In addition, the kidney weight ratio (expressed as a percentage of kidney weight to body weight) were also measured for comparison. At the end of chemotherapy (day 33), we found that both the CDDP alone (Group II, *p* < 0.0005) and the CDDP + MBs + US (Group III, *p* < 0.005) treatments resulted in significantly greater kidney weight ratios when compared to the control (Group I) treatment. By contrast, this ratio did not differ significantly for the CDDP-MBs (Group IV, *p* = 0.86) and CDDP-MBs + US (Group V, *p* = 0.87) treatments when compared to the control (Figure S3), suggesting that treatment with CDDP-loaded MBs prevented cisplatin-induced increases in kidney weight ratios. 

Taken together, these data highlight that conjugation of CDDP with MBs decreases CDDP cytotoxicity for tumor cells both in vitro and in vivo, but a similar antitumor effect is achieved when combined with US exposure. Likewise, the use of USMB to increase cell permeability and enhance CDDP uptake and apoptosis would result in a better tumor reduction outcome than is achieved with conventional CDDP alone.

## 3. Discussion

In this study, the microbubble shell was composed of human serum albumin (HSA). Numerous studies of cisplatin binding sites on albumin have shown that albumin interactions with CDDP may involve a binding site associated with the Cys34, Met329, Tyr148 or Tyr150, Asp375 or Glu376, and Met548 residues on HSA [20,21,22,23]. We generated CDDP-loaded MBs by first allowing CDDP to interact with HSA and then sonicating the preformed CDDP-albumin to generate MBs carrying CDDP. Because these MBs are generated from CDDP bound to albumin, CDDP can potentially be incorporated into the shell of the MB or into the core [24]. The CDDP-loaded MBs can also be prepared by first sonicating HSA into albumin MBs, followed by incubating the albumin MBs with cisplatin, but this preparation of CDDP attached to the MB surface may rely on a weak non-covalent interaction. We conducted a pilot experiment by incubating albumin MBs with CDDP for 17 h and found that the yield of CDDP-MBs was significantly low, with a dramatic decrease in the number, and the CDDP-MBs produced tended to precipitate at the bottom of the tube after centrifugation (Figure S4). A similar response has been reported for streptavidin prepared in microspheres, which bound only 50% of the amount of biotin normally bound by the native streptavidin [25]. The results from in vitro experiments, as shown in Figure 2D, also demonstrate that treating FaDu cells with CDDP alone or with MBs resulted in no significant difference in cell viability. This suggests that the influence of MBs on CDDP when administered together is negligible.

Unlike earlier reports where albumin mesospheres were directly used to load with CDDP and an organic solvent (DMSO) was used to enhance loading efficiency [19], our laboratory-made CDDP-MBs were prepared in normal saline solution and were composed of perfluorocarbon gas that was encapsulated in a serum albumin shell. In general, gas bubbles without a shell encapsulation are quite unstable in the bloodstream and may quickly dissolve; shell-free MBs could not even pass through the lung vasculature. Modern MBs usually consist of two parts: a core with an inert and low soluble gas, such as perfluorocarbon or perfluoropropane, and a stabilizing shell, such as phospholipids, proteins, and polymers. Engineering of the MB surface architecture and chemistry to reduce complement activation and phagocytosis [26,27] or directly modifying the lipid shell [28] helps to delay dissolution and ensure a longer half-life of the MBs in circulation. For some preparations, the prolonged circulation time may improve from many minutes up to 1 h [29]. In our current study, although the loading efficiency of CDDP onto the CDDP-MBs was limited to about 10%, the ultrasound-mediated destruction of these CDDP-MBs with an inert gas core preserved the effective cytotoxicity of CDDP without concerns about additional modifications or incorporation of other agents. Moreover, previous studies that evaluated the efficacy of CDDP aided by USMB were mostly focused on using the USMB to increase tumor uptake of CDDP by co-administration of MBs and CDDP [4,5,7,30,31]. To the best of our knowledge, this is the first study to prepare gas-filled and CDDP-carrying MBs for the application of USMB in cancer chemotherapy (Figure 6).

Although the platinum (Pt) of CDDP relies on its chloride ligands to enter cells when circulated in the bloodstream that contains high concentrations of chloride, only 10% of the introduced CDDP can enter into the cells, as 90% becomes bound to the plasma proteins [32]. Our results demonstrate that CDDP, when bound within albumin MBs, is rendered inactive and less toxic. The binding of CDDP to the MBs diminished its cytotoxicity, and CDDP loaded onto HSA also significantly decreased its cytotoxicity in FaDu cells (Figure S5). These results are consistent with previous studies that investigated the antitumor activity and toxicity of protein-bound CDDP complexes [33,34,35,36]. A phase I study of patients with head and neck cancers has shown that a cisplatin-albumin complex, either at a starting dose of 100 mg/m^2^ or a gradually escalated dose to 650 mg/m^2^, caused no significant nephrotoxicity or ototoxicity [36]. The CDDP-albumin complex is not as effective as conventional therapy, so, not surprisingly, the patients treated with the complex has a shorter median survival time when compared with patients receiving conventional CDDP therapy (109 days vs. 151 days). We attempted to overcome this drawback by diminishing the systemic side effects of chemotherapy using CDDP-loaded MBs, while enhancing their antitumor activity using US. The results presented here showed that nephrotoxicity is minimized by administration of CDDP-MBs when compared to conventional CDDP. Ultrasound treatment of the tumor following treatment with CDDP-MBs or CDDP + MBs resulted in similar or even better antitumor effects than were observed with conventional CDDP therapy. The combination of CDDP, MBs, and US gave the greatest tumor reduction through USMB-mediated cavitation effects on the tumor cells. Thus, US treatment gave CDDP enough momentum to travel within the tumor without affecting the cytotoxicity of free CDDP.

Data from our in vivo studies show that systemic biodistribution of Pt to relevant organs varied according to the treatment with different CDDP-based chemotherapies. The amount of Pt was significantly lower in the groups treated with CDDP-loaded MBs with or without ultrasound, when compared to the group receiving CDDP alone. One explanation may be that the larger CDDP-MB complex may slow the distribution of Pt into tissues when compared with the rapid distribution of the unbound CDDP molecule [36]. A previous study that explored the pharmacokinetics of both plasma protein-bound CDDP and unbound plasma CDDP showed that the terminal elimination half-life was 50 h for the bound form and 1.32 h for the unbound form when CDDP was administered as a 30-min intravenous infusion for five consecutive days [37]. Similarly, infusion of a CDDP-albumin complex at dose of 575 mg/m^2^ resulted in a prolonged terminal elimination half-life of about 250 h [36]. 

Several known risk factors, such as hypomagnesemia, cardiac disease, and hypoalbuminemia have been associated with cisplatin-induced nephrotoxicity. Cancer patients are known to have relatively decreased plasma albumin levels when compared to healthy subjects. Patients with lower albumin levels are prone to have a higher unbound fraction of plasma CDDP and a reduced CDDP half-life [38,39]. Hypoalbuminemia may also affect the peritubular oncotic pressure and in turn affect Pt excretion [40], thereby putting these patients at greater risk of nephrotoxicity. A recent in vitro study of plasma protein and distribution of Pt by Morris et al. [41] reported an increase in toxic CDDP-derived hydrolysis products and a decrease in protein bound Pt in the plasma of pediatric cancer patients with low serum albumin, when compared to plasma from healthy controls. Interestingly, an increase in the concentration of plasma serum albumin resulted in a decrease in the CDDP-derived hydrolysis products, suggesting that reinforcement of the plasma albumin concentration in cancer patients prior to CDDP treatment could be a simple strategy for alleviation of CDDP-induced toxic side effects. 

Patients treated with Pt analogues are also thought to be at high risk for developing hearing loss. Our experiments showed that the lowest Pt content of all analyzed organs was in the inner ear, and hearing assessment, as tested by the combined use of ABR and DPOAE, revealed no significant differences in the hearing thresholds and signal-to-noise ratio (SNR) of the distortion product (DP) measurements at frequencies from 4 kHz to 32 kHz among the treatment and control groups (Figure S6). Astolfi et al. [42] demonstrated that intraperitoneal injection of CDDP at a single high dose of 14 mg/kg or at 4.6 mg/kg/day for three consecutive days caused a hearing threshold shift in rats. We used a low dose of CDDP at 2 mg/kg twice a week, which might have effects that are too subtle for evaluating CDDP-induced hearing loss in mice. These inconsistencies suggest that differences in CDDP administration doses, the time intervals between courses, and the experimental models used could lead to different findings and outcomes in terms of ototoxicity.

The use of CDDP-bound MBs plus ultrasound treatment appears to have several advantages over existing chemotherapy methods, as the CDDP-MBs are less toxic than CDDP alone, the treatments are simple to perform, and most importantly, the CDDP-MBs are designed for systemic application. Local injection of CDDP and MBs, followed by US exposure of localized lymph nodes and tumors, has also demonstrated several benefits over conventional CDDP, but the pitfalls of this approach may include possible major blood vessel injury, restricted delivery to the injection site, and limited CDDP penetration due to the intratumoral interstitial pressure. Treatment of advanced stages of SCCHN also requires a more sophisticated systemic chemotherapy approach than a local approach in terms of extended locoregional control and ameliorating the rate of distant metastasis.

## 4. Materials and Methods

### 4.1. Animals and Study Design

**Figure 7:**
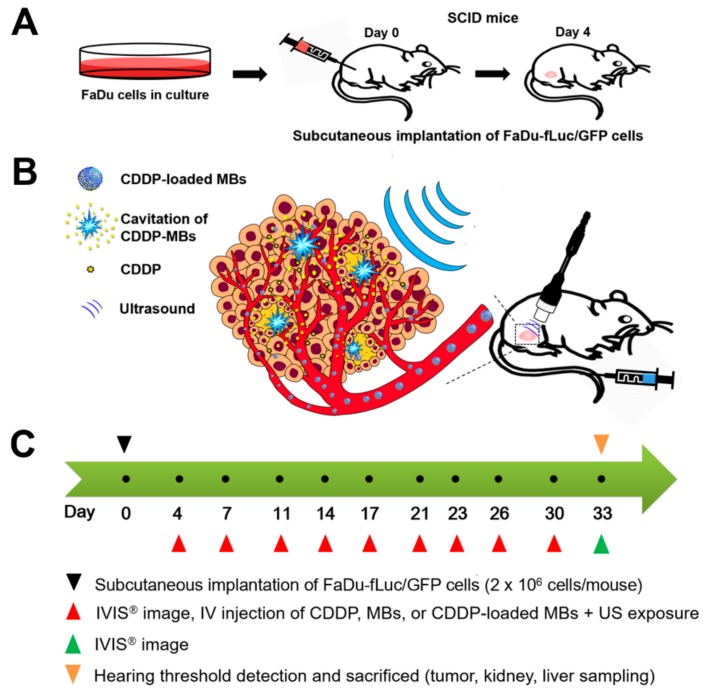
Scheme of animal study design. (**A**) SCID mice were subcutaneously implanted with FaDu-fLuc/GFP cells (2 × 10^6^ cells/mouse) into the right flank region on day 0; (**B**) On day 4, tumor xenograft formation and size were determined by IVIS^®^ bioluminescence images (version 4.4, Caliper Life Sciences, Alameda, CA, USA). Medication with CDDP-loaded MBs was given by intravenous injection via the tail vein, followed by ultrasound exposure directly on the tumor site; (**C**) Flow chart of the study design. CDDP = cisplatin; IV = intravenous; MBs = microbubbles; US = ultrasound.

Figure 7 illustrates the animal study design. Specific pathogen-free (SPF) severe combined immunodeficiency (SCID; CB17/lcr-*Prkdc*^scid^/lcrlcoCrlBltw) mice (female, aged 7–8 weeks) were obtained from BioLASCO (Taipei, Taiwan) and subsequently housed at the Animal Center of the National Defense Medical Center (Taipei, Taiwan) under SPF conditions. Experiments were conducted in accordance with the institutional guidelines and regulations, and the study protocol was approved by the Institutional Animal Care and Use Committee of the National Defense Medical Center, Taipei, Taiwan (ethic code: IACUC-16-328; permission date: 2 January 2017). 

The SCCHN tumor-bearing mouse model was established in the SCID mice after general anesthesia by isoflurane inhalation (2% in oxygen). On Day 0, the mice were injected with 100 µL of FaDu-fLuc/GFP cell suspension (2 × 10^6^ cells/mouse) subcutaneously into the right flank region using a 1 mL Monoject tuberculin syringe with a 25 G needle (Figure 7A). Tumor xenograft formation and size were monitored and evaluated by bioluminescence images (BLI) (Perkin Elmer, Waltham, MA, USA) on Day 4 by intraperitoneal injection of 150 mg/kg D-luciferin (Biosynth, Staad, Switzerland). After a 10 min distribution period, the tumors were quantified by measuring total photon flux per second. Because of the differences in tumor sizes, mice with similar tumor size were equally distributed and divided into five groups of 6–7 animals: Group I: saline control; Group II: CDDP; Group III: CDDP + MBs + US (1.4 × 10^8^ HSA microbubbles/mL) plus ultrasound (3 W/cm^2^, 30 s); Group IV: CDDP-MBs (1.4 × 10^8^ CDDP-loaded HSA microbubbles/mL); and Group V: CDDP-MBs + US (1.4 × 10^8^ CDDP-loaded HSA microbubbles/mL) plus ultrasound exposure (3 W/cm^2^, 30 s). All medications, including saline, CDDP, MBs, and CDDP-MBs, were given by intravenous injection in a volume of 100 μL via the tail vein on Days 4, 7, 11, 14, 17, 20, 23, 26, 30, and 33 (a total of 10 injections). Following each injection, the ultrasound probe was immediately applied for 30 s to the tumors of mice from Groups III and V, with jelly placed between the probe and the tumor (Figure 7B). 

The CDDP dose for each intravenous administration was 2 mg/kg, and the volume of CDDP-MBs required for an equivalent amount of CDDP was calculated. Before each chemotherapy session, the tumor volume was assessed by imaging with the IVIS system (Figure 7C). On Day 33, hearing was assessed using the auditory brainstem response (ABR) and distortion product otoacoustic emissions (DPOAEs). 

### 4.2. Preparation and Characterization of CDDP-Loaded MBs (CDDP-MBs)

**Figure 8:**
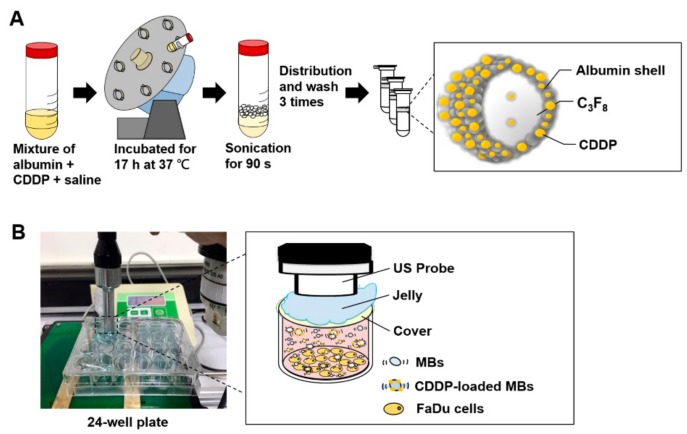
Schematic illustration of (**A**) the preparation of CDDP-loaded MBs and (**B**) optimization of ultrasound parameters in vitro.

The preparation of albumin MBs has been described in detail previously [43,44]. As illustrated in Figure 8A, our preparations of CDDP-loaded MBs (CDDP-MBs) were modified from existing methods [36], as follows: 6 mg/mL cisplatin (Sigma-Aldrich, St. Louis, MO, USA) in 2 mL saline was mixed with 140 mg/mL human serum albumin solution (Octapharma, Vienna, Austria), incubated in the dark at 37 °C, and placed on a vortex mixer (30 rpm) for 17 h. Saline was then added to make 10 mL, followed by a 90 s sonication with perfluorocarbon (C_3_F_8_) gas using a sonicator (Branson Ultrasonics Co., Danbury, CT, USA). The sample was then centrifuged in 1 mL aliquots at 1200 rpm for 2 min (Thermo Fisher Science, Bremen, Germany). The lower layer was removed and 1 mL of physiological saline (pH 7.4, 0.9% sodium chloride) was added for re-centrifugation. These steps were repeated three times to eliminate the free (unbound) cisplatin. Aliquots of the CDDP-MBs were stored at 4 °C. 

The size distribution of CDDP-MBs in the solution was measured by dynamic light scattering (Nanoparticle Analyzer, Horiba, Kyoto, Japan), and the bubble number was measured with a MultiSizer III device (Beckman Coulter, Fullerton, CA, USA) using a 30-μm aperture probe with a measurement boundary ranging from 0.6 to 20 μm.

The CDDP-MBs and MBs were filtered with a 5-μm syringe filter (Sartorius, Goettingen, Germany), and then fixed with 0.25% glutaraldehyde (Sigma-Aldrich, St. Louis, MO, USA). The morphology and structure of the hardened CDDP-MBs and MBs were investigated using a light microscope (Primo Star; Carl Zeiss, Jena, Germany) equipped with a digital camera (AxioCam ERc 5 s; Zeiss) and a scanning electron microscope (JFC-1300, JEOL, Tokyo, Japan) after platinum coating for 20 min at 20 mA [45].

### 4.3. Quantitation of CDDP-Loaded MBs (CDDP-MBs) by ICP-MS

The CDDP release and its loading efficiency were quantitatively analyzed by adding 3 mL concentrated nitric acid (Ultrex II, J.T. Baker, MT, USA) to microwave digestion vessels containing 100 μL CDDP-MBs and digesting in a CEM Mars 5 Microwave Accelerated Reaction System (CEM Corp., Matthews, NC, USA). The parameters of the microwaving cycle were as follows: power 240 W; temperature 180 °C; pressure 170 psi; and hold time 15 min, then cooling down to room temperature. The digested samples were diluted with ddH_2_O to 25 mL and analyzed by inductively coupled plasma mass spectrometry (ICP-MS) (XSeries 2 spectrometer; Thermo Fisher, Hemel Hempstead, UK) with the typical normal mode conditions: extraction voltage was typically −35 V, Rf Power 510 W, focus voltage 4.5 V, and nebulizer gas flow rate 1.04 L/min. Dwell times were 10 ms, with 100 sweeps per replicate and five replicates per sample. The platinum (Pt) standard for ICP (TraceCERT^®^, 1000 mg/L Pt in hydrochloric acid, Sigma-Aldrich, Milan, Italy) was used for the preparation of working standard solutions [19]. The CDDP content (μg per mL of CDDP-MBs) of various compositions of CDDP and albumin, as determined by using ICP-MS, ranged from 308–403 μg/mL.

### 4.4. Tumor Cell Line and Culture

The FaDu human pharyngeal squamous carcinoma cell line (American Type Culture Collection, HTB-43, Manassas, VA, USA) was maintained in Eagle’s Minimum Essential Medium (EMEM; Biological Industries, Beit Haemek, Israel) supplemented with 10% fetal bovine serum (FBS), 1 mM sodium pyruvate, 1× non-essential amino acids (NEAA), 100 U/mL penicillin, and 100 μg/mL streptomycin (Biological Industries) and incubated at 37 °C in a humidified 5% CO_2_ atmosphere. Cells were routinely subcultured to 70–80% confluence and experiments were performed with cells from passage 6–12.

### 4.5. Lentiviral Transduction of the FaDu Cell Line

The growth of the implanted FaDu tumor cells was monitored noninvasively by transducing the cells with a bicistronic lentiviral vector encoding a firefly luciferase (Fluc) transgene under the cytomegalovirus (CMV) promoter, as well as a green fluorescent protein (GFP) transgene driven by the SV40 promoter, using a puromycin resistance gene as a selectable marker (titer: 2 × 10^9^ IU/mL, Tools Biotechnology, Inc., New Taipei City, Taiwan). This lentiviral vector was produced by cloning the Fluc complementary DNA sequence into the pLenti-GIII-CMV-GFP-2A-Puro lentiviral vector (Applied Biological Materials Inc., Richmond, BC, Canada). The day before transduction, cells were seeded in six-well plates at 1 × 10^5^ cells per well. On the day of transduction, the medium was replaced with 2 mL fresh EMEM containing 10 µg/mL polybrene (Sigma, St. Louis, MO, USA), and lentiviral vector was added to the cells at a multiplicity of infection (MOI) of 10. After 48 h of culture, the cell culture medium was replaced with fresh medium containing 10 µg/mL puromycin (InvivoGen, San Diego, CA, USA). The medium was replaced with fresh puromycin-containing medium every 3–4 days until resistant colonies were identified. 

The GFP expressed in the surviving cells were checked by fluorescence microscopy, while the intracellular firefly luciferase activity was measured by adding 150 μg/mL D-luciferin-containing medium (Biosynth) and imaging using the IVIS system. The surviving cells were trypsinized and further cultured with a limited dilution method to select a single colony for further culture and to establish a stable FaDu cell line expressing both luciferase and GFP reporter genes (FaDu-fLuc/GFP). 

### 4.6. Bioluminescence Imaging

Tumor growth of head and neck carcinoma in living animals was evaluated using non-invasive bioluminescence imaging (Perkin Elmer, Waltham, MA, USA) of SCID mice with implanted xenograft tumors from FaDu-fLuc/GFP cells. Before imaging, the mice were anesthetized with isoflurane and then intraperitoneally injected with D-luciferin potassium salt (150 mg/kg; Biosynth, Staad, Switzerland) in Dulbecco's phosphate-buffered saline (DPBS). After 10 min, each mouse was placed in a ventral decubitus position, and bioluminescence imaging was performed using a cryogenically cooled IVIS imaging system coupled with a data acquisition computer running Living Image software. A digital grayscale animal image was acquired, followed by acquisition and overlay of a pseudocolor image representing the spatial distribution of the detected photon counts emerging from active luciferase within the tumor. The luminescence signal intensity was reported as radiance (photons/sec/cm^2^/steradian) with a color bar. The tumor volumes were quantified by measuring the total photon counts per second (p/s) within the region of interest. Tumor growth was monitored on Day 4 after FaDu cell implantation, and tumors were imaged twice a week for the duration of the experiment.

### 4.7. Optimization of Ultrasound Parameters for Ultrasound-Mediated MB Destruction 

As illustrated in Figure 8B, per well in a 24-well plate was filled with MBs (1.4 × 10^7^ MBs/mL, about 4 mL) and no air bubbles were trapped when the cover was placed. An ultrasound instrument (ST2000V, Nepagene, Japan) equipped with a 10-mm diameter probe, with center frequency 1 MHz and duty cycle of 50%, was used for irradiation. The probe was placed directly on the 24-well cover, with jelly used as a transducer medium. The ultrasonic parameters for MB destruction in vitro were established by investigating the following settings for ultrasound exposure: 1 W/cm^2^, 30 s; 1 W/cm^2^, 60 s; 1 W/cm^2^, 30 s twice; 1 W/ cm^2^, 30 s, 4 times; or 1 W/cm^2^, 30 s, 6 times. After ultrasound exposure, the MB solution in each well was diluted 10 times and was then imaged using the US animal imaging system (Prospect, S-Sharp Corp., Taipei, Taiwan). Images were processed with custom MATLAB programs (The Math Works, Natick, MA, USA) to evaluate the destruction efficiency by calculating the difference in the gradient strength of the MB images before and after ultrasound exposure. 

The in vitro effects of ultrasound-mediated MB destruction on cancer cells were evaluated in FaDu cells seeded in a 24-well plate at 2 × 10^4^ cells/well overnight. The next day, about 4 mL of MBs (1.4 × 10^7^ MBs/mL) were used to fill each well, followed by treatments with ultrasound as already described. After the ultrasound exposure, the MB solution was replaced with EMEM and the cells were allowed to grow for 48 h. The WST-1 assay was then used to assess cell viability.

### 4.8. Cell Viability Assay

The medium was replaced with EMEM containing 10% WST-1 (water-soluble tetrazolium salt) assay agent (Roche Applied Science, Mannheim, Germany), and the cells were incubated for another 2 h. Viable cells catalyzed the production of formazan dye by mitochondrial reductase, and the amount of dye was quantified by measuring the absorbance at 450 nm using a Multi-Mode Reader (Synergy 2, BioTek Instruments, Winooski, VT, USA). Wells without cells were used as the blank. Cell viability was calculated according to the following formula: Cell viability (%) = cells (sample)/cells (control) × 100. 

### 4.9. Sonication Efficiency of MBs Evaluated by Agarose Phantoms

The in vivo sonication effects on MBs were evaluated by high-frequency US imaging using an US animal imaging system (Prospect, S-Sharp Corporation, New Taipei City, Taiwan) in tissue-mimicking agarose phantoms, as described previously [46]. Briefly, a 2% agarose square-column (10 × 20 × 20 mm^3^) phantom was constructed with a 2 × 2 × 20 mm^3^ chamber at its center to load 400 μL of 1.4 × 10^7^ microbubbles/mL, followed by sonication using a 10-mm diameter and 1-MHz US transducer (ST2000V, Nepagene, Ichikawa, Japan). The acoustic intensity was set at 1, 2, or 3 W/cm^2^, for 30 s, with a duty cycle of 50% and a pulse repetition period (PRP) of 250 ms. The region of interest (ROI) was drawn over the entire MB-loaded chamber in two-dimensional imaging planes by the operator, the dynamic range was set at 50 dB, and the average pre- and post-sonication image intensities were measured in B-mode images. 

### 4.10. High Frequency US Imaging for Accessing In Vivo MB Destruction

The sonication efficiency in the agarose phantom, or the adherent MBs in the xenograft tumors in mice and the subsequent MB destruction mediated by US, were monitored by high frequency US imaging using a commercial small-animal US imaging system (Prospect; S-Sharp Corporation, New Taipei City, Taiwan) [43]. The ultrasound images were obtained using a 40 MHz central frequency transducer with axial and lateral resolutions of 30 and 60 μm, respectively. The axial and lateral fields of view were 20 and 20 mm, respectively. Real-time imaging was performed at a frame rate of 20 Hz (corresponding to a temporal resolution of 50 ms). Two-dimensional B-mode image planes were acquired with optimization of the gain and the time-gain compensation settings, which were kept constant throughout the experiments. 

Before conducting the imaging experiments, the mice were anesthetized with 2% isoflurane and the hair covering the region of tumor was removed. The transducer of the high frequency US imaging device was fixed onto a railing system and the acoustic focus was centered at the level of the subcutaneous tumor xenografts, with the imaging plane aligned in the center of the tumor. Upon injection of the MBs into the tail vein, a therapeutic US exposure was applied at an intensity of 3 W/cm^2^ for 30 s. Images representing the MBs in the tumor were displayed by the imaging system as green overlays on the brightness mode anatomic images. 

### 4.11. In Vitro Cytotoxicity of CDDP-MBs in the FaDu Cell Line

FaDu cells were seeded at a density of 2 × 10^4^ cells/well in 24-well plates, cultured in humidified air with 5% CO_2_ at 37 °C overnight, and then divided into three groups (*n* = 5 per group) for incubation with different combinations of CDDP, MBs, and CDDP-MBs for 48 h, with or without ultrasound exposure (1 W/cm^2^, 30 s for six times), as follows: Group 1: CDDP only (0, 1, 5 and 10 μM), Group 2: CDDP (0, 1, 5 and 10 μM) with MBs (1.4 × 10^7^ MBs/mL), and Group 3: CDDP-MBs (CDDP loading content: 0, 1, 5 and 10 μM in 1.4 × 10^7^ MBs/mL). After a 48-h incubation, the cell viability was assessed with the WST-1 assay. 

### 4.12. Biodistribution of Cisplatin

After the experimental treatments, the xenograft tumor-bearing mice in each group were injected intraperitoneally with barbiturate (150 mg/kg) and sacrificed by cervical dislocation. Subsequently, liver, kidneys, inner ear, and tumors were excised, immediately frozen in liquid nitrogen, and stored at −80 °C until analysis. For platinum distribution analysis, 100 mg of the sampled tissues were added to 3 mL HNO_3_ and digested in a microwave oven. Analyses were carried out using the ICP-MS XSeries 2 instrument. All analyses were conducted on five replicates per sample.

### 4.13. Sample Preparation for Histopathological Examination

The xenograft tumor mass and kidney excised from each sacrificed mouse in each group were fixed and processed for paraffin embedding. The paraffin-embedded tissue cassettes were mounted onto a rotary microtome, sectioned to a thickness of approximately 5 µm, and stained with hematoxylin and eosin (H&E). The sections were examined with an Olympus B×50 brightfield/fluorescence microscope (Olympus Corp., Tokyo, Japan) equipped with a digital camera (Olympus DP74, Olympus Corp., Tokyo, Japan). The digital photomicrographs were processed with the cellSens Standard 1.17 (Olympus) software.

### 4.14. Auditory Brainstem Response Recording

Each animal’s auditory function was assessed by recording the ABRs, as previously described [47]. In brief, the mice were anesthetized and kept warm with a heating pad in a sound-attenuating chamber. Subdermal needle electrodes were inserted at the vertex (positive), below the pinna of the ear (negative), and at the back (ground) of the mice. Specific stimuli (clicks and 8, 16, 20, 24, and 32 kHz tone bursts) were generated using SigGen software (Tucker-Davis Technologies, Alachua, FL, USA) and delivered to the external auditory canal. The average responses to 1024 stimuli at each frequency were obtained by reducing the sound intensity in 5 dB steps until the threshold was reached. The resulting ABR thresholds were defined as the lowest intensity at which a reproducible deflection in the evoked response trace could be recognized.

### 4.15. Distortion Product Otoacoustic Emission Measurements

The DPOAEs were measured at the following center frequency (F_C_): 8, 16, 20, 24, and 32 kHz with a Real-time Signal Processing System (Tucker-Davis Technologies, Gainesville, FL, USA). Two simultaneous continuous pure tones, F_1_ and F_2_, were calculated using the center frequency, where F_1_ was F_C_*0.909 and F_2_ was F_C_*1.09. This yielded a frequency of primary 1, named Tone 1, and primary 2, named Tone 2, that were geometrically centered about F_C_. The two primary tones were presented at the same intensity (L1 = L2 = 65 dB SPL) and at a frequency ratio (F_2_/F_1_) of 1.2 [48]. The primary tones produced by two separate speakers (EC1 close-field speakers; Tucker-Davis Technologies) were introduced into the animal's ear canal. DPOAE recordings were made with a low-noise microphone (ER 10B; Etymotic Research, Elk Grove Village, IL, USA) and averaged 512 times at each frequency. The peak of the cubic difference distortion product (2F_1_ − F_2_) at different center frequencies (F_C_) was accepted as a DPOAE if it was 3 dB above the noise floor, and the difference was referred to as the signal-to-noise ratio (SNR). 

### 4.16. Terminal Deoxynucleotidyl Transferase dUTP Nick End Labeling (TUNEL) Assay

Paraffin-embedded xenograft tumor sections were dewaxed in xylene and rehydrated through a graded series of ethanol and double-distilled water, followed by a PBS wash. The TUNEL assays were performed according to the instructions provided with the In Situ Cell Death Detection Kit, POD (Roche, Basel, Switzerland). Deparaffinized slides were incubated with 3% H_2_O_2_ in methanol for 10 min to block endogenous peroxidase activity and rinsed with PBS. The tissues were permeabilized with 0.1% Triton X-100 in 0.1% sodium citrate for 10 min. After PBS washes, the tissues were blocked with blocking buffer (Tris-HCl, 0.1 M/3% BSA/20% normal bovine serum) for 30 min at room temperature and incubated with the TUNEL reaction mixture for 60 min at 37 °C in a humidified atmosphere in the dark. After PBS Tween-20 (PBST) washing, the tissues were stained with Converter-POD for an additional 30 min and washed with PBST. Labeled apoptotic cells were identified by treating the slides with DAB substrate for 10 min. The nuclei were counterstained with hematoxylin by incubating the sections at room temperature for 5 min. Apoptotic cells stained yellow and nuclei stained blue. Slides were examined with an Olympus B×50 brightfield/fluorescence microscope (Olympus Corp., Tokyo, Japan) equipped with a digital camera (Olympus DP74). Digital photomicrographs were processed with the cellSens Standard 1.17 (Olympus) software. The color intensity of images was analyzed using the open-source Fiji software (ImageJ IHC toolbox, version 1.51, National Institutes of Health, Bethesda, MD, USA). The color intensity of the control group was regarded as 1.0, and the relative color intensity of the other groups was evaluated against the control.

### 4.17. Statistical Analysis

Statistical analysis was performed using a two-tailed Student’s *t*-test for comparison of the means between two groups and two-way ANOVA with Tukey’s multiple comparison test for the means for more than three groups. Graphpad Prism software was used for all statistical analysis (version 6.0 GraphPad Software Inc., San Diego, CA, USA). Results were expressed as the mean ± standard error of the mean (SEM). Differences were considered significant at *p* < 0.05.

## 5. Conclusions

Cisplatin (CDDP) chemotherapy for patients with SCCHN is inherently associated with dose-limiting toxic side effects. The results presented here showed that the use of CDDP-loaded MBs together with US enhanced the antitumor effects in areas given localized ultrasound exposure while sparing other organs and suppressing toxicity, mainly nephrotoxicity and ototoxicity. This approach therefore holds potential for use in future head and neck cancer therapy.

## Figures and Tables

**Table 1:** Composition, diameter, number, and CDDP loading efficiency of CDDP-loaded albumin MBs.

Concentration of Albumin (mg/mL)	Concentration of CDDP (mg/mL)	CDDP Content (µg/mL of CDDP-MBs)	Loading Efficiency (%)	MB Diameter (μm)	MB Concentration (×10^8^ Bubbles/mL)
132	-	-	-	1.02 ± 0.11	1.40 ± 2.22
132	6	391.3 ± 4.25	9.78 ± 0.25	1.70 ± 0.73	0.71 ± 0.56
140	6	403.5 ± 4.52	10.09 ± 0.23	2.05 ± 0.61	1.09 ± 1.31
150	6	308 ± 24.3	7.70 ± 1.21	2.45 ± 0.45	1.05 ± 0.36

Values are expressed as mean ± SEM (*n* = 5); CDDP = cisplatin; MBs = microbubbles.

## Supplementary Materials

The following are available online at http://www.mdpi.com/2072-6694/10/9/311/s1, Figure S1: Comparison of loading efficiency of cisplatin (CDDP) in human serum albumin (HSA) microbubbles. To achieve the optimal CDDP concentration for high CDDP loading efficiency of the CDDP-HSA, various concentrations of CDDP (1, 2, 4, 6, and 12 mg/mL) were mixed with albumin (140 mg/mL). A CDDP dose-dependent efficacy was observed between 1 and 6 mg/mL, whereas no significant difference was noted between 6 and 12 mg/mL CDDP; Figure S2: Quantification of tumor apoptosis after various CDDP-based chemotherapies, as confirmed by Terminal Deoxynucleotidyl Transferase dUTP Nick End Labeling (TUNEL) assays and analyzed using ImageJ software. The color intensity of the control group was set as 1.0, and the relative color intensity of the treatment groups was evaluated and expressed as the mean values ± SEM. * *p* < 0.05; ** *p* < 0.01. CDDP + MBs + US = the combinations of CDDP, microbubbles, and ultrasound exposure; CDDP-MBs = CDDP-loaded microbubbles; CDD − MBs + US = CDDP-loaded microbubbles plus ultrasound exposure; Figure S3: Analysis of the kidney weight ratio at the end of treatment (day 33). The values for this ratio for each chemotherapy group were 0.75 ± 0.01 (Group I), 1.0 ± 0.06 (Group II), 0.94 ± 0.04 (Group III), 0.79 ± 0.01 (Group IV), and 0.79 ± 0.03 (Group V). Values are expressed as mean ± SEM (*n* = 4). * *p* < 0.05, ** *p* < 0.005, # *p* < 0.0005 (one-way ANOVA with Tukey’s multiple comparison test). NS = not significant; Figure S4: Incubation of MBs with CDDP to generate CDDP-loaded MBs (CDDP-MBs). The control group was MBs incubated in phosphate-buffered saline. After incubation for 17 h, CDDP-MBs precipitated from the rest of the mixture after centrifugation, and a marked drop was noted in the number of MBs (right panel) compared with control (left panel). MB = microbubble. Scale bar = 10 μm; Figure S5: Comparison of the in vitro toxicity of CDDP, CDDP-loaded MBs, and CDDP-loaded HSA in the FaDu cell line. After administration of various equivalent doses of CDDP treatment for 48 h, the percentages of viable cells were evaluated by WST-1 analysis. The results are expressed as the mean ± standard error of the mean (SEM), with *n* = 4 for each bar. * *p* < 0.05; ** *p* < 0.005. CDDP = cisplatin; MB = microbubble; HSA = human serum albumin; Figure S6: Hearing assessment in mice after 33 days of various CDDP-based chemotherapies. (A) The auditory brainstem response (ABR) thresholds in each group. (B) Signal-to-noise ratios (SNRs) of the cubic difference distortion product (2F_1_–F_2_) at different center frequencies (F_C_) were obtained in each group. The results are expressed as the mean ± standard error of the mean (SEM), with *n* = 4 for each bar.
