# Supplementary material for: Insonation of Systemically Delivered Cisplatin-Loaded Microbubbles Significantly Attenuates Nephrotoxicity of Chemotherapy in Experimental Models of Head and Neck Cancer
Source: Cancers (Basel). 2018 Sep 5;10(9):311. doi: 10.3390/cancers10090311 (PMC6162676; doi:10.3390/cancers10090311)
Supplement: Supplementary file 1 [file cancers-10-00311-s001.pdf]

Supplementary Materials: Insonation of Systemically Delivered Cisplatin-Loaded Microbubbles Significantly Attenuates Nephrotoxicity of Chemotherapy in Experimental Models of Head and Neck Cancer

Hang-Kang Chen, Shu-Mei Zhang, Junn-Liang Chang, Hsin-Chien Chen, Yi-Chun Lin, Cheng-Ping Shih, Huey-Kang Sytwu, Mei-Cho Fang, Yuan-Yung Lin, Chao-Yin Kuo, Ai-Ho Liao, Yueng-Hsiang Chu and Chih-Hung Wang

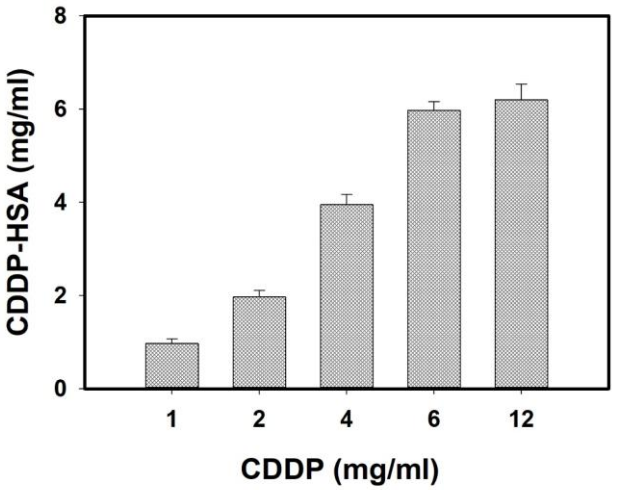

Figure S1. Comparison of the loading efficiency of cisplatin (CDDP) in human serum albumin (HSA) microbubbles. To achieve the optimal CDDP concentration for high CDDP loading efficiency of the CDDP-HSA, various concentrations of CDDP (1, 2, 4, 6, and 12 mg/mL) were mixed with albumin (140 mg/mL). A CDDP dose-dependent efficacy was observed between 1 and 6 mg/mL, whereas no significant difference was noted between 6 and 12 mg/mL CDDP.

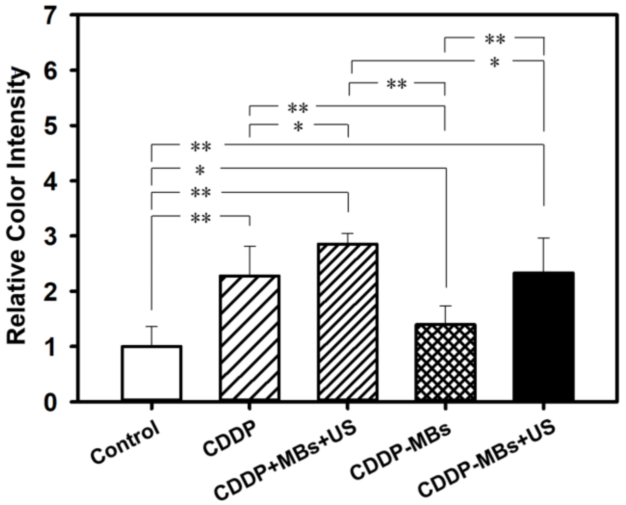

Figure S2. Quantification of tumor apoptosis after various CDDP-based chemotherapies, as confirmed by Terminal Deoxynucleotidyl Transferase dUTP Nick End Labeling (TUNEL) assays and analyzed using ImageJ software. The color intensity of the control group was set as 1.0, and the relative color intensity of the treatment groups was evaluated and expressed as the mean values ± SEM. * $p < 0.05$; ** $p < 0.01$. CDDP+MBs+US = the combinations of CDDP, microbubbles, and

ultrasound exposure; CDDP-MBs = CDDP-loaded microbubbles; CDDP-MBs+US = CDDP-loaded microbubbles plus ultrasound exposure.

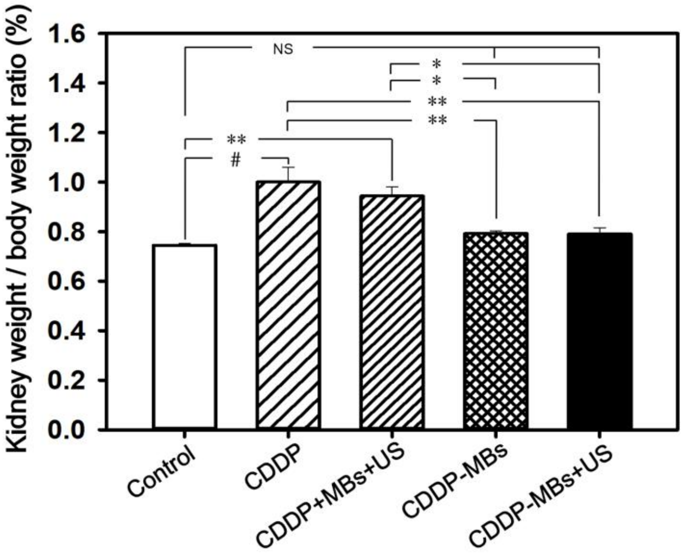

Figure S3. Analysis of the kidney weight ratio at the end of treatment (day 33). The values for this ratio for each chemotherapy group were 0.75 ± 0.01 (Group I), 1.0 ± 0.06 (Group II), 0.94 ± 0.04 (Group III), 0.79 ± 0.01 (Group IV), and 0.79 ± 0.03 (Group V). Values are expressed as mean ± SEM (n = 4). * $p < 0.05$, ** $p < 0.005$, # $p < 0.0005$ (one-way ANOVA with Tukey's multiple comparison test). NS = not significant.

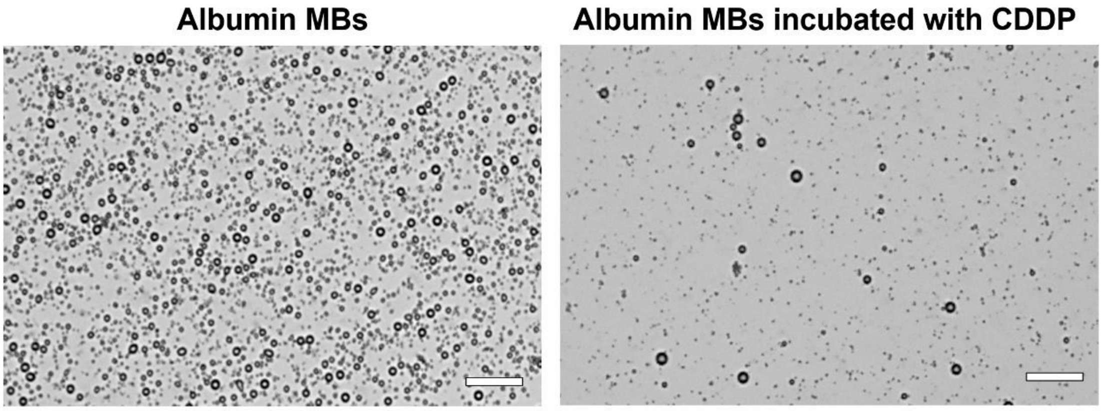

Figure S4. Incubation of MBs with CDDP to generate CDDP-loaded MBs (CDDP-MBs). The control group was MBs incubated in phosphate buffered saline. After incubation for 17 h, CDDP-MBs precipitated from the rest of the mixture after centrifugation, and a marked drop was noted in the number of MBs (right panel) compared with control (left panel). MB = microbubble. Scale bar = 10 μm.

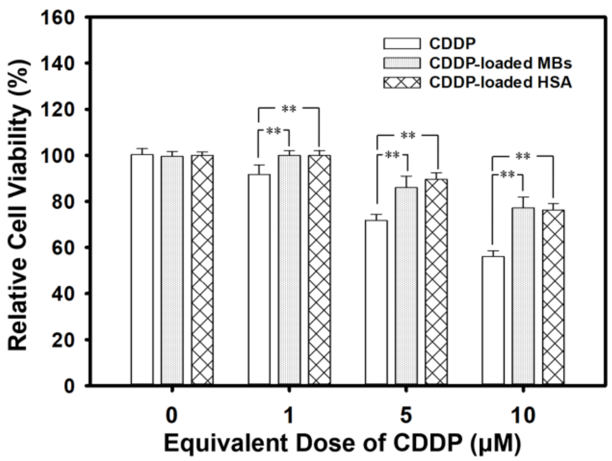

Figure S5. Comparison of the in vitro toxicity of CDDP, CDDP-loaded MBs, and CDDP-loaded HSA in the FaDu cell line. After administration of various equivalent doses of CDDP treatment for 48 h, the percentages of viable cells were evaluated by WST-1 analysis. The results are expressed as the mean ± standard error of the mean (SEM), with $n = 4$ for each bar. * $p < 0.05$; ** $p < 0.005$. CDDP = cisplatin; MB = microbubble; HSA = human serum albumin.

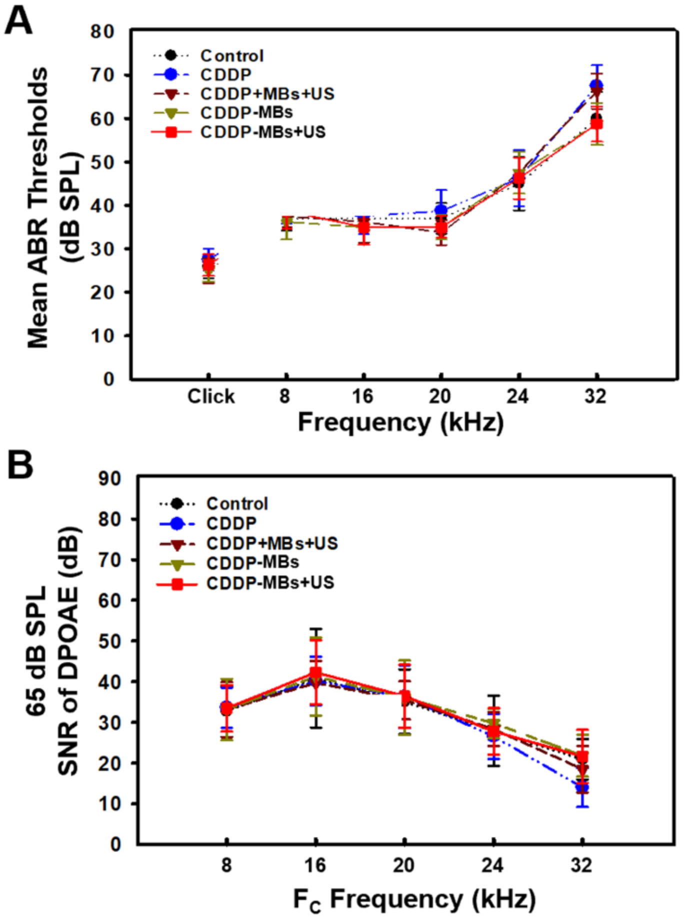

Figure S6. Hearing assessment in mice after 33 days of various CDDP-based chemotherapies. (**A**) The auditory brainstem response (ABR) thresholds in each group. (**B**) Signal-to-noise ratios (SNRs) of the cubic difference distortion product ($2F_1-F_2$) at different center frequencies (F_C) were obtained in each group. The results are expressed as the mean ± standard error of the mean (SEM), with $n = 4$ for each bar.
